# Synthesis and Antitumor Activities of Phenanthrene-Based Alkaloids

**DOI:** 10.3390/molecules14125042

**Published:** 2009-12-07

**Authors:** Songtao Li, Li Han, Liang Sun, Dan Zheng, Jiang Liu, Yingbo Fu, Xueshi Huang, Zhanyou Wang

**Affiliations:** 1 Department of Natural Products Chemistry, Pharmaceutical School, China Medical University, Shenyang 110001, China; 2 Laboratory of Cell Engineering and Cell Therapy, College of Basic Medical Science, China Medical University, Shenyang 110001, China

**Keywords:** PBTs, synthesis, cytotoxicity

## Abstract

A series of phenanthrene-based tylophorine derivatives (PBTs) were synthesized and their cytotoxic activities against the H460 human large-cell lung carcinoma cell line were evaluated. Among these compounds, *N*-(3-hydroxy-2,6,7-tri-methoxyphenanthr-9-ylmethyl)-l-prolinol (**5a**), and *N*-(3-hydroxy-2,6,7-trimethoxy-phenanthr-9-ylmethyl)-l-valinol (**9**) exhibited good activities, with IC_50_ values of 11.6 and 6.1 μM, respectively.

## 1. Introduction 

Phenanthroindolizidine alkaloids have been of considerable interest as anticancer agents because of their exceptionally potent antitumor activity [1]. Based on recent studies, the molecular target of phenanthroindolizidine analogues may be novel and different from the targets of known anticancer drugs [2]. Over the past decades, the synthesis and biological activities of varied 2,3,6,7-functionalized phenanthroindolizidine alkaloids have been reported. The substituents mainly include functionalities such as hydroxyl, methoxyl, and hydrogen [3,4,5]. The structure activity relationships of phenanthroindolizidine alkaloids were summarized by Gao *et al*. in 2007 [3]. The degree of cytotoxicity was dependent on the type and pattern of substitution on the phenanthrene ring. In 2006, Wei *et al*. [6] found that phenanthrene-based tylophorine derivatives (PBTs), which were phenanthroindolizidine derivatives resulting from opening of the indolizidine ring, exhibited significant cytotoxic activity. Further work from the same group resulted in the synthesis of a series of PBTs with different substituents at C-9, which indicated that a five- or six-carbon distance between the nitrogen and a terminal polar substituent in the C-9 chain is quite favorable for cytotoxic activity [7]. They also reported that antitumor activities of PBTs were induced by inhibition of the activation of Akt and NF-κB signaling pathway in tumor cells [8,9]. 

**Scheme 1:**
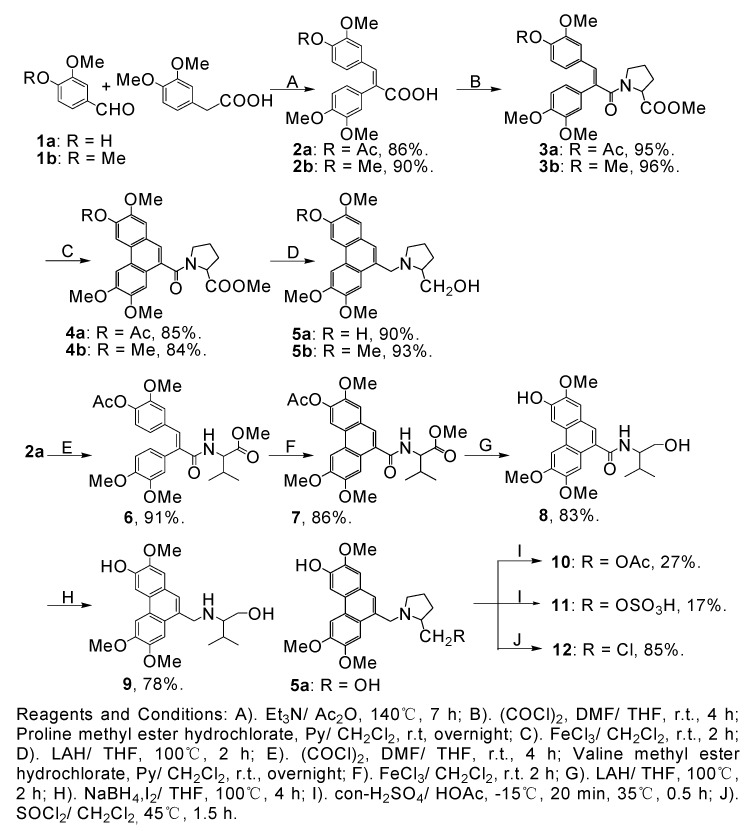
The synthetic routes to PBTs.

As part of our studies on antitumor agents derived from natural products, we have designed and synthesized a series of PBT analogues to investigate the effects of oxygenic functional groups on the phenanthrene ring and C-9 side chain on cytotoxic activity. The present paper reports the synthesis of these PBTs (Scheme 1) and evaluation of their cytotoxic activities against the H460 human large-cell lung carcinoma cell line *in vitro* (Table 1).

## 2. Results and Discussion

### 2.1. Chemical synthesis

The synthetic routes to the PBTs are depicted in Scheme 1. Perkin condensation [10] involving 4-hydroxy-3-methoxy- or 3,4-dimethoxybenzaldehyde and 3,4-dimethoxyphenylacetic acid in the presence of Ac_2_O and Et_3_N afforded the cinnamic acid derivatives **2a** (R = Ac) or **2b** (R = Me) [11,12,13,14]. By treatment with oxalyl chloride [15], the acids **2a** or **2b** gave an acid chloride intermediate which could be converted to the corresponding aromatic amides **3a** (R = Ac) or **3b** (R = Me) [10] by using l-proline methyl ester hydrochloride. Through oxidation-coupling with ferric trichloride [11] as the catalyst, the amides were efficiently cyclized to deliver 9-amido-substituted phenanthrenes **4a** (R = Ac) and **4b** (R = Me) [10,16], respectively. Finally, the two amides were reduced with LiAlH_4_ [17] to give **5a** (R = OH) and **5b** (R = Me) [18,19]. By treatment with concentrated H_2_SO_4_/HOAc [20] or SOCl_2_/CH_2_Cl_2_ [21]_,_ **5a** was converted to **10** **and 11,** or **12**. Compound **7** was synthesized from **2a** and l-valine methyl ester hydrochloride following the same synthetic route as described for **4a.**

In our synthetic route, the key step is the reduction of the aromatic amides with aluminum and boron hydrides. In the reduction process we tried LiAlH_4_/THF [17], NaBH_4_-MeSO_3_H/DMSO [22] and NaBH_4_-I_2_/THF [23] systems to reduce compound **7**. First, reduction with the NaBH_4_-MeSO_3_H/DMSO system did not afford the target product, but rather a series of small amounts of unidentified products. Then, by using LiAlH_4_ as the catalyst, the 3-acetoxyl on the phenanthrene ring and the ester group in the amido side chain of **7** were reduced to hydroxyl and hydroxymethyl groups, respectively, as shown for compound **8**, but the carbonyl linkage was not reduced. Finally, under the catalysis of NaBH_4_-I_2_/THF system, **8** was successfully reduced to **9** in a satisfactory yield of 78%. However, compounds **4a** and **4b** were completely reduced using the LiAlH_4_/THF system in one step to give **5a** and **5b** in consistently high yields of 90% and 93%, respectively. 

### 2.2. Biological activity

The synthesized PBTs were screened for *in vitro* cytotoxic activity against the H460 human large-cell lung carcinoma cell line with an MTT assay procedure [24]. Adriamycin was used as the reference compound. Table 1 summarizes the structures of **5a**, **5b**, **8**-**12** and their cytotoxicities (IC_50_) against the H460 cell line. All of them exhibited diminished cytotoxic activities to some extent, in comparison to their parent natural products (e.g., tylophorine, IC_50_ 0.5~1.7 μM) [25]_._

The results indicate that cytotoxicities of PBTs were influenced by the presence of oxygenic functional substituents on the phenanthrene, and amino acid side chains at the C-9 position of phenanthrene and the linkage between the nitrogen and the phenanthrene. Replacing the 3-methoxyl on the phenanthrene skeleton with a hydroxyl increased the cytotoxic activity, as shown in the comparison of **5b** (3-methoxyl, IC_50_ 53.8 μM) and **5a** (3-hydroxyl, IC_50_ 11.6 μM). Thus, a hydroxyl at the phenanthrene C-3 is quite favorable for cytotoxic activity. In the prolinol side chain, when the terminal hydroxyl was converted to an acetate ester, sulfate ester and chloride, the cytotoxic activities were significantly diminished, it may relate to the removal of the terminal hydrogen bond effect on the assumed biological target [7]. Conserving the C-3 hydroxyl substitution on the phenanthrene skeleton of **5a** and opening the pentacyclic prolinol resulted in a more active compound **9** (IC_50_ 6.1μM). This result indicates that changing the cyclic side chain to an acyclic one may increase the flexibility of the C-9 side chain, which favors attaining an optimal conformation for binding to an assumed biological target. The amide derivative **8** exhibited very low activity (IC_50_ 68.1μM), in agreement with the report of Wei *et al*. [6].

## 3. Experimental

### 3.1. General

Melting points of the synthesized compounds were determined on a Digital Melting Point Apparatus XT4A and are uncorrected. IR spectra were recorded with a Perkin-Elmer model 298 spectrometer (KBr). 1D NMR spectra were recorded on Bruker ARX-300 or Bruker AV-600 spectrometers. ESIMS were recorded with a Finnigan LCQ mass spectrometer and EIMS were measured by an Agilent/HP HP5973 and 6890 GC/MSD. Column chromatography was carried out on silica gel (200-300 mesh, Qingdao Marine Chemical Ltd., Qingdao, China). The MTT assay was recorded on a microplate reader (KHB ST-360, SH Kehua Laboratory System Co., Ltd., Shanghai, China). Thin-layer chromatography (TLC) was performed on TLC silica gel 60 F254 plates (0.5 mm, Merck). Chemicals from Sinopharm Chemical Reagent Co. were used without further purification.

### 3.2. Preparation of PBT derivatives

#### 3.2.1. Synthesis and spectroscopic data of 4-acetoxy-α-(3,4-dimethoxyphenyl)-3-methoxycinnamic acid (**2a**) and 3,4-dimethoxy-α-(3,4-dimethoxyphenyl)cinnamic acid (**2b**) 

A mixture of 4-hydroxy-3-methoxybenzaldehyde (**1a**, 0.15 g, 1 mmol), 3,4-dimethoxyphenylacetic acid (0.19 g, 1 mmol), acetic anhydride (2 mL) and triethylamine (0.3 mL) was heated to reflux at 140 °C for 7 h using a CaCl_2_ tube. Then the mixture was cooled to room temperature, diluted with water (10 mL) and refluxed at 120 °C for 30 min. The cooled mixture was extracted with EtOAc (3 × 50 mL). The combined organic extracts were washed with saturated brine (150 mL), dried over anhydrous Na_2_SO_4_, filtered, concentrated *in vacuo* and the residue recrystallized from methanol to afford **2a** (0.32 g, 86%) as pale-yellow crystals. m.p. 166-168 °C; ^1^H-NMR (300 MHz, CDCl_3_) δ: 2.28 (s, 3H, CH_3_CO), 3.47 (s, 3H, OCH_3_), 3.82 (s, 3H, OCH_3_), 3.90 (s, 3H, OCH_3_), 6.67 (d, 1H, *J* = 1.5 Hz, Ar-H), 6.79 (d, 1H, *J* = 1.5 Hz, Ar-H), 6.83 (dd, 1H, *J* = 8.2, 1.5 Hz, Ar-H), 6.84 (dd, 1H, *J* = 8.1, 1.5 Hz, Ar-H), 6.91 (d, 1H, *J* = 8.1 Hz, Ar-H), 6.92 (d, 1H, *J* = 8.2 Hz, Ar-H), 7.87 (s, 1H, C=CH); IR (KBr) cm^-1^ *ν*: 3421, 3062, 2964, 2839, 1764, 1674, 1619, 1516; EIMS m/z: 372 [M]^+^ (C_20_H_20_O_7_), 330 (100%) [M-acyl]^+^. Compound **2b** (0.31 g, 90%) was synthesized as a deep yellow solid from 3,4-dimethoxy-benzaldehyde (**1b**, 0.17 g, 1 mmol) and 3,4-dimethoxyphenylacetic acid (0.19 g, 1 mmol) in the same synthetic route as that for **2a**. m.p. 217-219 °C; ^1^H-NMR (300 MHz, CDCl_3_) δ: 3.47 (s, 3H, OCH_3_), 3.81 (s, 3H, OCH_3_), 3.84 (s, 3H, OCH_3_), 3.89 (s, 3H, OCH_3_), 6.55 (d, 1H, *J* = 1.8 Hz, Ar-H), 6.70 (d, 1H, *J* = 1.8 Hz, Ar-H), 6.72 (d, 1H, *J* = 8.5 Hz, Ar-H), 6.84 (dd, 1H, *J* = 8.2, 1.8 Hz, Ar-H), 6.85 (dd, 1H, *J* = 8.5, 1.8 Hz, Ar-H), 6.92 (d, 1H, *J* = 8.2 Hz, Ar-H), 7.86 (s, 1H, C=CH); IR (KBr) cm^-1^ *ν*: 3448, 3043, 2938, 2835, 1668, 1593, 1510; ESI MS m/z: [M-H]^-^ 343 (C_19_H_20_O_6_).

#### 3.2.2. Synthesis and spectroscopic data of methyl N-[4-acetoxy-α-(3,4-dimethoxyphenyl)-3-methoxy)-cinnamyl]pyrrolidine-2-carboxylate (**3a**) and methyl N-[3,4-dimethoxy-α-(3,4-dimethoxyphenyl)-cinnamyl]pyrrolidine-2-carboxylate (**3b**) 

To a stirred solution of **2a** (0.74 g, 2 mmol) and DMF (0.1 mL) in anhydrous THF (10 mL), oxalyl chloride (2 mL, 22.4 mmol) in anhydrous THF (2 mL) was added dropwise. After the addition was complete, the mixture was stirred at room temperature for 4 h. Excess oxalyl chloride was removed under reduced pressure. The yellow residue was dissolved in anhydrous CH_2_Cl_2_ (20 mL), to which was added dropwise a solution of l-proline methyl ester hydrochloride (0.35 g, 2.1 mmol) and pyridine (1 mL, 12.4 mmol) in anhydrous CH_2_Cl_2_ (4 mL) with stirring. The mixture was stirred overnight at room temperature. Then 1 N HCl (15 mL) was added to the reaction system with vigorous stirring to quench the reaction. The organic layer was seperated and the aqueous layer was extracted with CH_2_Cl_2_ (2 × 30 mL). The combined organic extracts were washed with saturated brine (90 mL) and dried over anhydrous Na_2_SO_4_. After filtration and evaporation *in vacuo*, the residue was purified by column chromatography on silica gel (hexane/EtOAc, 1:1.25 v/v) to afford **3a** (0.92 g, 95%) as a colorless solid; m.p. 53-55 °C; ^1^H-NMR (300 MHz, CDCl_3_) δ: 1.81-1.94 (m, 3H overlapped, 1.5×CH_2_), 2.23 (m, 1H, 0.5×CH_2_), 2.26 (s, 3H, CH_3_CO), 3.30 (m, 2H, CH_2_N), 3.53 (s, 3H, OCH_3_), 3.73 (s, 3H, OCH_3_), 3.76 (s, 3H, OCH_3_), 3.85 (s, 3H, OCH_3_), 4.55 (m, 1H, CHN), 6.76-6.92 (m, 7H overlapped, 7×Ar-H); IR (KBr) cm^-1^ *ν*: 3041, 2954, 2836, 1764, 1745, 1633, 1600, 1513; EIMS m/z: 483 [M]^+^ (C_26_H_29_NO_8_), 441 [M-acyl]^+^, 424 [M-COOMe]^+^, 327 [M-CO-C_6_H_10_NO_2_]^+^, 285 (100%) [M-CO-C_6_H_10_NO_2_ -acyl]^+^. Compound **3b** (1.27 g, 96%) was synthesized as a light yellow solid from **2b** (1 g, 2.9 mmol) and l-proline methyl ester hydrochloride (0.49 g, 3.0 mmol) by the same synthetic route as that for **3a**; m.p. 44-46 °C; ^1^H-NMR (300 MHz, CDCl_3_) δ: 1.81-1.98 (m, 3H overlapped, 1.5×CH_2_), 2.24 (m, 1H, 0.5×CH_2_), 3.34 (m, 2H, CH_2_N), 3.57 (s, 3H, OCH_3_), 3.78 (s, 3H, OCH_3_), 3.85 (s, 3H, OCH_3_), 3.88 (s, 3H, OCH_3_), 4.56 (m, 1H, CHN), 6.70-6.95 (m, 7H overlapped, 7×Ar-H); IR (KBr) cm^-1^ *ν*: 3047, 2953, 2836, 1743, 1632, 1600, 1514; EIMS m/z: 455 [M]^+^ (C_25_H_29_NO_7_), 327 [M-C_6_H_10_NO_2_]^+^, 299 (100%) [M-CO-C_6_H_10_NO_2_]^+^. 

#### 3.2.3. Synthesis and spectroscopic data of methyl N-(3-acetoxy-2,6,7-trimethoxyphenanthr-9-ylcarbonyl)pyrrolidine-2-carboxylate (**4a**) and methyl N-(2,3,6,7-tetramethoxyphenanthr-9-ylcarbonyl)-pyrrolidine-2-carboxylate (**4b**)

To a solution of **3a** (0.48 g, 1 mmol) in anhydrous CH_2_Cl_2_ (15 mL) was added anhydrous FeCl_3_ (0.68 g, 4.3 mmol), and the mixture was stirred at room temperature for 2 h under nitrogen. Then 1 N HCl (1 mL) diluted with saturated brine (10 mL) was added to the stirred reaction system. The organic layer was seperated and the aqueous layer was extracted with CH_2_Cl_2_ (2 × 30 mL). The combined organic extracts were washed with saturated brine (80 mL) and dried over anhydrous Na_2_SO_4_. After filtration and evaporation, the residue was purified by column chromatography on silica gel (hexane/EtOAc, 1:1.5 v/v) to afford **4a** (0.41 g, 85%) as an off-white solid; m.p. 229-231 °C; ^1^H-NMR (600 MHz, CDCl_3_) δ: 1.88 (m, 1H, 0.5×CH_2_), 1.95 (m, 1H, 0.5×CH_2_), 2.09 (m, 1H, 0.5×CH_2_), 2.37 (m, 1H, 0.5×CH_2_), 2.43 (s, 3H, CH_3_CO), 3.22 (m, 1H, 0.5×CH_2_N), 3.36 (m, 1H, 0.5×CH_2_N), 3.84 (s, 3H, CH_3_O), 3.97 (s, 3H, CH_3_O), 4.00 (s, 3H, CH_3_O), 4.01 (s, 3H, CH_3_O), 4.84 (dd, 1H, *J* = 8.4, 4.8 Hz, CHN), 7.29 (s, 1H, Ar-H), 7.59 (s, 1H, Ar-H), 7.70 (s, 1H, Ar-H), 7.77 (s, 1H, Ar-H), 8.14 (s, 1H, Ar-H); IR (KBr) cm^-1^ *ν*: 3083, 2953, 2836, 1763, 1743, 1631, 1508; ESI MS m/z: [M+H]^+^ 482 (C_26_H_27_NO_8_). Compound **4b** (1.33 g, 84%) was synthesized as a light yellow solid from **3b** (1.6 g, 3.5 mmol) and anhydrous FeCl_3_ (2.27 g, 14 mmol) by a similar synthetic route as that of **4a**; m.p. 200-202 °C; ^1^H-NMR (300 MHz, CDCl_3_) δ: 1.77-1.98 (m, 2H overlapped, CH_2_), 2.06 (m, 1H, 0.5×CH_2_), 2.32 (m, 1H, 0.5×CH_2_), 3.22 (m, 1H, 0.5×CH_2_N), 3.36 (m, 1H, 0.5×CH_2_N), 3.81 (s, 3H, CH_3_O), 3.98 (s, 3H, CH_3_O), 4.06 (s, 3H, CH_3_O), 4.09 (s, 6H, 2×CH_3_O), 4.81 (m, 1H, CHN), 7.14 (s, 1H, Ar-H), 7.52(s, 1H, Ar-H), 7.64 (s, 1H, Ar-H), 7.73 (s, 1H, Ar-H), 7.74 (s, 1H, Ar-H); IR (KBr) cm^-1^ *ν*: 3077, 2952, 2836, 1741, 1628, 1509; EIMS m/z: 453 [M]^+^ (C_25_H_27_NO_7_), 325 (100%) [M-C_6_H_10_NO_2_]^+^.

#### 3.2.4. Synthesis and spectroscopic data of N-(3-hydroxy-2,6,7-trimethoxyphenanthr-9-ylmethyl)-l-prolinol (**5a**) and N-(2,3,6,7-tetramethoxyphenanthr-9-ylmethyl)-l-prolinol (**5b**)

To a stirred, ice-salt bath cooled solution of **4a** (3.1 g, 6.4 mmol) in anhydrous THF (100 mL) was added LiAlH_4_ (1.3 g, 39 mmol) in one portion. After being stirred for 30 min kept at 0 °C, the mixture was heated to reflux at 100 °C for 2 h using a CaCl_2_ tube. To the cooled mixture were added ice and saturated NaHCO_3_ solution (100 mL). After stirring for 10 min, the THF layer was separated and the aqueous layer was extracted with THF (2 × 50 mL). The combined organic extracts were washed with saturated brine (200 mL) and dried over anhydrous Na_2_SO_4_. After filtration and evaporation, the residue was purified by column chromatography on silica gel (CH_2_Cl_2_/MeOH, 20:1 v/v) to afford **5a** (2.3 g, 90%) as an off-white solid. m.p. 178-180 °C; ^1^H-NMR (600 MHz, CDCl_3_) δ: 1.70 (m, 1H, 0.5×CH_2_), 1.74 (m, 1H, 0.5×CH_2_), 1.87 (m, 1H, 0.5×CH_2_), 2.02 (m, 1H, 0.5×CH_2_), 2.46 (m, 1H, 0.5×CH_2_N), 2.95 (m, 1H, 0.5×CH_2_N), 2.97 (m, 1H, CHN), 3.52 (d, 1H, *J* = 10.8 Hz, 0.5×CH_2_O), 3.74 (d, 1H, *J* = 12.6 Hz, 0.5×ArCH_2_N), 3.79 (d, 1H, *J* = 10.8 Hz, 0.5×CH_2_O), 4.03 (s, 3H, CH_3_O), 4.08 (s, 6H, 2×CH_3_O), 4.47 (d, 1H, *J* = 12.6 Hz, 0.5×ArCH_2_N), 7.18 (s, 1H, Ar-H), 7.50 (s, 1H, Ar-H), 7.57 (s, 1H, Ar-H), 7.82 (s, 1H, Ar-H), 7.95 (s, 1H, Ar-H); IR (KBr) cm^-1^ *ν*: 3388, 3050, 2953, 2839, 1617, 1513; ESI MS m/z: 398 [M+H]^+^ (C_23_H_27_NO_5_). Compound **5b** (0.84 g, 93%) was synthesized as a white solid from **4b** (1 g, 2.2 mmol) and LiAlH_4_ (0.7 g, 21 mmol) by the same synthetic route as that described for **5a**; m.p. 217-218 ^o^C; ^1^H-NMR (300 MHz, CDCl_3_) δ: 1.57-1.67 (m, 2H overlapped, CH_2_), 1.79 (m, 1H, 0.5×CH_2_), 1.91 (m, 1H, 0.5×CH_2_), 2.38 (m, 1H, 0.5×CH_2_N), 2.80 (m, 1H, 0.5×CH_2_N), 2.87 (m, 1H, CHN), 3.42 (dd, 1H, *J* = 10.5, 2.4 Hz, 0.5×CH_2_O), 3.65 (d, 1H, *J* = 12.6 Hz, 0.5×ArCH_2_N), 3.68 (dd, 1H, *J* = 10.5, 3.6 Hz, 0.5×CH_2_O), 3.96 (s, 3H, CH_3_O), 4.00 (s, 3H, CH_3_O), 4.02 (s, 3H, CH_3_O), 4.04 (s, 3H, CH_3_O), 4.39 (d, 1H, *J* = 12.6 Hz, 0.5×ArCH_2_N), 7.12 (s, 1H, Ar-H), 7.44 (s, 1H, Ar-H), 7.52 (s, 1H, Ar-H), 7.70 (s, 1H, Ar-H), 7.74 (s, 1H, Ar-H); IR (KBr) cm^-1^ *ν*: 3423, 3050, 2958, 2836, 1617, 1510; ESI MS m/z: 412 [M+H]^+^ (C_24_H_29_NO_5_).

#### 3.2.5. Synthesis and spectroscopic data of N-[4-acetoxy-α-(3,4-dimethoxyphenyl)-3-methoxy-cinnamyl]-l-valine methyl ester (**6**) 

Compound **6** (1.90 g, 91%) was synthesized as a colorless solid from **2a** (1.6 g, 4.3 mmol) and l-valine methyl ester hydrochloride (0.75 g, 4.5 mmol) by a similar synthetic route as that for **3a**; m.p. 46-48 °C; ^1^H-NMR (300 MHz, CDCl_3_) δ: 0.76 (d, 3H, *J* = 6.9 Hz, CH_3_), 0.91 (d, 3H, *J* = 6.8 Hz, CH_3_), 2.13 (m, 1H, CH), 2.27 (s, 3H, CH_3_CO), 3.48 (s, 3H, OCH_3_), 3.71 (s, 3H, OCH_3_), 3.84 (s, 3H, OCH_3_), 3.93 (s, 3H, OCH_3_), 4.65 (brdd, 1H, *J* = 8.7, 4.9, CHN), 6.10 (d, 1H, *J* = 8.7 Hz, NH), 6.62 (d, 1H, *J* = 1.7 Hz, Ar-H), 6.75 (dd, 1H, *J* = 8.1, 1.7 Hz, Ar-H), 6.81 (d, 1H, *J* = 1.7 Hz, Ar-H), 6.85 (d, 1H, *J* = 8.2 Hz, Ar-H), 6.88 (dd, 1H, *J* = 8.2, 1.7 Hz, Ar-H), 6.98 (d, 1H, *J* = 8.1 Hz, Ar-H), 7.77 (s, 1H, C=CH); IR (KBr) cm^-1^ *ν*: 3420, 3050, 2961, 2836, 1767, 1740, 1670, 1616, 1513; EIMS m/z: 485 [M]^+^ (C_26_H_31_NO_8_), 443 (100%) [M-acyl]^+^, 328[M-CO-C_6_H_12_NO_2_]^+^, 285[M-acyl-CO-C_6_H_12_NO_2_]^+^.

#### 3.2.6. Synthesis and spectroscopic data of N-(3-acetoxy-2,6,7-trimethoxyphenanthr-9-ylcarbonyl)-l-valine methyl ester (**7**) 

Compound **7** (335 mg, 86%) was synthesized as an off-white solid from **6** (390 mg, 0.80 mmol) and anhydrous FeCl_3_ (0.53 g, 3.24 mmol) using the same synthetic route as that for **4a**; m.p. 205-206 °C; ^1^H-NMR (300 MHz, CDCl_3_) δ: 1.02 (d, 3H, *J* = 6.9 Hz, CH_3_), 1.12 (d, 3H, *J* = 6.8 Hz, CH_3_), 2.36 (m, 1H, CH), 2.43 (s, 3H, CH_3_CO), 3.83 (s, 3H, OCH_3_), 3.98 (s, 3H, OCH_3_), 4.01 (s, 3H, OCH_3_), 4.09 (s, 3H, OCH_3_), 4.93 (brdd, 1H, *J* = 8.8 Hz, 4.9Hz, CHN), 6.64 (d, 1H, *J* = 8.8 Hz, NH), 7.29 (s, 1H, Ar-H), 7.73 (s, 1H, Ar-H), 7.76 (s,1H, Ar-H), 7.79 (s, 1H, Ar-H), 8.11(s, 1H, Ar-H); IR (KBr) cm^-1^ *ν*: 3288, 3050, 2961, 2834, 1760, 1742, 1635, 1598, 1508; ESI MS m/z: 482[M-H]^-^ (C_26_H_28_NO_8_).

#### 3.2.7. Synthesis and spectroscopic data of N-(3-hydroxy-2,6,7-trimethoxyphenanthr-9-ylcarbonyl)-l-valinol (**8**)

A 100 mL dry three-necked flask was charged with a stirred suspension of LiAlH_4_ (61 mg, 1.6 mmol) in anhydrous THF (15 mL) kept at −10~0 °C for 10 min. Compound **7** (150 mg, 0.31 mmol) dissolved in anhydrous THF (15 mL) was added dropwise to the suspension with stirring at −10~0 °C. After the addition was complete, stirring was continued for 10 min, then the warmed reaction system was refluxed at 100 °C for 2 h using a CaCl_2_ tube. To the cooled mixture were added ice and saturated NaHCO_3_ solution (30 mL). After being stirred for 10 min, the THF layer was separated and the aqueous layer was extracted with CH_2_Cl_2_ (2 × 30 mL). The combined organic extracts were washed with saturated brine (90 mL), dried over anhydrous Na_2_SO_4_, filtered and concentrated *in vacuo*. The residue was purified by column chromatography on silica gel (CH_2_Cl_2_/MeOH, 30:1 v/v) to afford **8** (106 mg, 83%) as a white solid; m.p.182-183 °C; ^1^H-NMR (300 MHz, DMSO) δ: 0.94 (d, 3H, *J* = 6.8 Hz, CH_3_), 0.99 (d, 3H, *J* = 6.8 Hz, CH_3_), 1.98 (m, 1H, CH), 3.56 (m, 2H, CH_2_O), 3.81 (s, 3H, OCH_3_), 3.92(s, 3H, OCH_3_), 3.96(m, 1H, CHN), 3.98(s, 3H, OCH_3_), 7.43 (s, 1H, Ar-H), 7.69 (s, 1H, Ar-H), 7.70 (s, 1H, Ar-H), 7.85 (s, 1H, Ar-H), 7.98 (s, 1H, Ar-H), 8.12(d, 1H, *J* = 9.15 Hz, NH); IR (KBr) cm^-1^ *ν*: 3296, 3050, 2957, 2835, 1620, 1593, 1508; ESI MS m/z: 412 [M-H]^-^ C_23_H_26_NO_6_ . 

#### 3.2.8. Synthesis and spectroscopic data of N-(3-hydroxy-2,6,7-trimethoxyphenanthr-9-ylmethyl)-l-valinol (**9**)

Compound **8** (41 mg, 0.1 mmol) dissolved in anhydrous THF (5 mL) was added dropwise to a stirred solution of NaBH_4_ (20 mg, 0.5 mmol) in anhydrous THF (20 mL) kept at −5~0 °C. Then I_2_ (59 mg, 0.23 mmol) dissolved in anhydrous THF (7 mL) was added dropwise to the stirred reaction system at −5~0 °C over 2 h. After the addition was complete, the mixture was warmed to room temperature and heated to reflux at 100 °C for 4 h. Then to the cooled mixture, 1 N HCl (8 mL) was added dropwise with stirring to quench the excess hydride. After the gas evolution ceased, saturated NaHCO_3_ solution (8 mL) was added dropwise to neutralize the excess HCl. The THF layer was separated and dried over anhydrous Na_2_SO_4_. After filtration, a solution of BF_3_·Et_2_O (2 mL) in THF (5 mL) was added dropwise to the filtrate, then saturated NaHCO_3_ solution (10 mL) was added to dissociate the target amine. The THF layer was separated, and the aqueous layer was extracted with THF (2 × 30 mL). The combined extracts were washed with saturated brine (100 mL) and dried over anhydrous Na_2_SO_4_. After filtration and evaporation *in vacuo*, the residue was purified by column chromatography on silica gel (CH_2_Cl_2_/MeOH, 15:1 v/v) to afford **9** (31 mg, 78%) as an off-white solid; m.p. 224-225 °C; ^1^H-NMR (300 MHz, DMSO) δ: 0.91 (d, 3H, *J* = 6.7 Hz, CH_3_), 0.96 (d, 3H, *J* = 6.7 Hz, CH_3_), 2.15 (m, 1H, CH), 2.98 (m, 1H, CHN), 3.60 (m, 1H, 0.5×OCH_2_), 3.73 (m, 1H, 0.5×OCH_2_), 3.92 (s, 3H, OCH_3_), 3.93 (s, 3H, OCH_3_), 3.98 (s, 3H, OCH_3_), 4.45 (m, 2H, ArCH_2_N), 7.33 (s, 1H, Ar-H), 7.59 (s, 1H, Ar-H), 7.68 (s, 1H, Ar-H), 7.86 (s, 1H, Ar-H), 7.97(s, 1H, Ar-H); IR (KBr) cm^-1^ *ν*: 3423, 3050, 2927, 2836, 1618, 1508; ESI MS m/z: [M+H]^+^ 400 (C_23_H_29_NO_5_).

#### 3.2.9. Synthesis and spectroscopic data of [N-(3-hydroxy-2,6,7-trimethoxyphenanthr-9-ylmethyl)-pyrrolidin-2-yl]methyl acetate (**10**) and [N-(3-hydroxy-2,6,7-trimethoxyphenanthr-9-ylmethyl)pyrro-lidin-2-yl]methyl hydrogen sulfate (**11**)

To a dry 50 mL three-necked flask charged with **5a** (1 g, 2.52 mmol) was added glacial acetic acid (5 mL) at −15 °C. The mixture was stirred for 5 min. Then frozen concentrated sulfuric acid (10 mL) was added dropwise to the stirred system at −15 °C. After the addition was complete, stirring was continued for 20 min. Then the solution was warmed to room temperature and gently heated to 35 °C. Thirty min later, the reaction mixture was cooled to room temperature, poured into water (100 mL) and adjusted to pH 10 with 1N KOH solution. The resulting mixture was extracted with EtOAc (3 × 80 mL). The combined extracts were washed with saturated brine (250 mL), dried over anhydrous Na_2_SO_4_, filtered and concentrated *in vacuo*. The residue was purified by column chromatography on silica gel (CH_2_Cl_2_/MeOH, 15:1 v/v) to afford **10** and **11.** Compound **10** (0.3 g, 27%), a white solid; m.p. 136-137 °C; ^1^H-NMR (600 MHz, CDCl_3_) δ: 1.66 (m, 3H overlapped, 1.5×CH_2_), 2.01 (m, 1H, 0.5×CH_2_), 2.06 (s, 3H, CH_3_CO), 2.35 (m, 1H, 0.5×CH_2_N), 2.84 (m, 1H, 0.5×CH_2_N), 2.96 (m, 1H, CHN), 3.68 (d, 1H, *J* = 13.2 Hz, 0.5×ArCH_2_N), 4.03 (s, 3H, OCH_3_), 4.05 (s, 3H, OCH_3_), 4.09 (s, 3H, OCH_3_), 4.13 (m, 1H, 0.5×CH_2_O), 4.29 (m, 1H, 0.5×CH_2_O), 4.61 (d, 1H, *J* = 13.2 Hz, 0.5×ArCH_2_N), 7.18 (s, 1H, Ar-H), 7.49 (s, 1H, Ar-H), 7.81 (s, 1H, Ar-H), 7.84 (s, 1H, Ar-H), 7.96 (s, 1H, Ar-H); IR (KBr) cm^-1^ *ν*: 3430, 3050, 2953, 2834, 1736, 1618, 1509; ESI MS m/z: 438 [M-H]^-^, 440 [M+H]^+^ (C_25_H_29_NO_6_). Compound **11** (0.2 g, 17%), a white solid; m.p. 216-218 °C; ^1^H-NMR (600 MHz, DMSO) δ: 1.78 (m, 1H, 0.5×CH_2_), 1.80 (m, 1H, 0.5×CH_2_), 2.02 (m, 1H, 0.5×CH_2_), 2.23 (m, 1H, 0.5×CH_2_), 3.10 (m, 1H, 0.5×CH_2_N), 3.35 (m, 1H, 0.5×CH_2_N), 3.94 (s, 3H, OCH_3_), 4.00 (s, 3H, OCH_3_), 4.03 (s, 3H, OCH_3_), 4.14 (m, 2H, CH_2_O), 4.34 (d, 1H, *J* = 13.2 Hz, 0.5×ArCH_2_N), 4.57 (m, 1H, CHN), 5.33 (d, 1H, *J* = 13.2 Hz, 0.5×ArCH_2_N), 7.35 (s, 1H, Ar-H), 7.56 (s, 1H, Ar-H), 7.82 (s, 1H, Ar-H), 7.91 (s, 1H, Ar-H), 8.01 (s, 1H, Ar-H); IR (KBr) cm^-1^ *ν*: 3423, 3050, 2924, 2850, 1624, 1514; ESI MS m/z: 476 [M-H]^-^, 478 [M+H]^+^ (C_23_H_27_NO_8_S). 

#### 3.2.10. Synthesis and spectroscopic data of N-(3-hydroxy-2,6,7-trimethoxyphenanthr-9-ylmethyl)-2-chloromethylpyrrolidine (**12**)

A 250 mL dry three-necked flask fitted with a dropping funnel and a condenser was charged with **5a** (2 g, 5.04 mmol) and anhydrous CH_2_Cl_2_ (70 mL). The condenser was fitted with a trap to remove the vapors of hydrogen chloride and sulfur dioxide. Freshly distilled SOCl_2_ (5 mL) in anhydrous CH_2_Cl_2_ (30 mL) was added dropwise to the stirred system at 0 °C. After the addition was complete, the mixture was warmed to room temperature and heated to reflux at 45 °C for 1.5 h, after which the reaction system was cooled to room temperature and adjusted to pH 10 with saturated Na_2_CO_3_ solution (100 mL) at 0 °C. The organic layer was separated and the aqueous layer was extracted with CH_2_Cl_2_ (2 × 50 mL), the combined extracts were washed with saturated brine (200 mL) and dried over anhydrous Na_2_SO_4_. After filteration and evaporation *in vacuo*, the residue was purified by column chromatography on silica gel (PE/EtOAc, 3:1 v/v) to afford **12** (1.78 g, 85%) as an off-white solid; m.p. 184-185 °C; ^1^H-NMR (300 MHz, CDCl_3_) δ: 1.67 (m, 2H overlapped, CH_2_), 1.82 (m, 1H, 0.5×CH_2_), 2.12 (m, 1H, 0.5×CH_2_), 2.26 (m, 1H, 0.5×CH_2_N), 2.41 (m, 1H, 0.5×CH_2_Cl), 2.72 (m, 1H, 0.5×CH_2_N), 3.08 (m, 1H, 0.5×CH_2_Cl), 3.83 (d, 1H, *J* = 13.2 Hz, 0.5×ArCH_2_N), 3.97 (d, 1H, *J* = 13.2 Hz, 0.5×ArCH_2_N), 3.98 (m, 1H, CHN), 4.03 (s, 3H, OCH_3_), 4.05 (s, 3H, OCH_3_), 4.10 (s, 3H, OCH_3_), 7.17 (s, 1H, Ar-H), 7.44 (s, 1H, Ar-H), 7.82 (s, 1H, Ar-H), 7.86 (s, 1H, Ar-H), 7.96 (s, 1H, Ar-H); IR (KBr) cm^-1^ *ν*: 3442, 3050, 2947, 2832, 1619, 1508; ESI MS m/z: 416 [M+H]^+^ (C_23_H_26_ClNO_4_). 

### 3.3. Cytotoxic assay

Cytotoxicities were determined by MTT method [24] using human large-cell lung carcinoma cell line (H460) grown in RPM1-1640 medium plus 10% heat-inactived fetal bovine serum. The assays were performed in 96-well microtiter plates. Compounds **5a, 5b, 8-12** were dissolved in DMSO and diluted to six different concentrations (10, 3.3, 1.0, 0.33, 0.10, and 0.033 mM) respectively, and each solution was ten-fold diluted to six different concentrations using culture medium (1.0, 0.33, 0.10, 0.033, 0.010, and 0.0033 mM), then 10 µL of each solution was added to 90 µL (about 5,000 cells) culture medium wells. After incubation at 37 °C for 72 hours, 10 µL of MTT (5 mg/mL) was added to each well and incubated for four hours, and then liquid in the wells was removed. DMSO (150 µL) was added to each well. The absorbance was recorded on a microplate reader at wavelength of 590 nm, and the IC_50_ was defined as 50% reduction of absorbance in the control assay. Compound **5a, 5b, 8-12** showed cytotoxicity against human tumor cell line H460 with IC_50_ value of 11.6, 53.8, 68.1, 6.1, 46.8, 53.4 and 62.9 μM, respectively. Adriamycin, with an IC_50_ value of 1.72 μM, was used as positive control.

## 4. Conclusions 

In conclusion, we have synthesized a series of PBT analogues by a facile route and evaluated their *in vitro* cytotoxic activities against human large-cell lung carcinoma cell line (H460). On this basis, the SAR analysis and potential antitumor activities of these alkaloids are under investigation. Compounds **5a** and **9** with a substitution of hydroxyl at position 3 and a hydroxyl at C-9 side chain terminus were the most effective cytotoxic compounds. These results may be helpful for the design of future PBT antitumor reagents, and offer potential application in the discovery of antitumor drugs.

## Figures and Tables

**Table 1:**
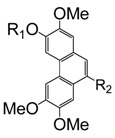
The structures of **5a**, **5b**, **8**-**12** and their cytotoxicities against H460 cell line.

Compound	R_1_	R_2_	IC_50_^a^ (μM)
**5a**	H	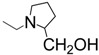	11.6
**5b**	OMe	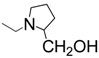	53.8
**8**	H	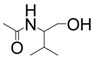	68.1
**9**	H	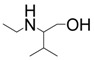	6.1
**10**	H	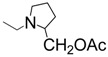	46.8
**11**	H	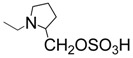	53.4
**12**	H	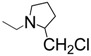	62.9

Adriamycin used as positive control, IC_50_ = 1.72 μM.
